# Supplementary material for: Milli-scale cellular robots that can reconfigure morphologies and behaviors simultaneously
Source: Nat Commun. 2022 Jul 18;13:4156. doi: 10.1038/s41467-022-31913-0 (PMC9293897; doi:10.1038/s41467-022-31913-0)
Supplement: Supplementary file 1 — Supplementary Information [file 41467_2022_31913_MOESM1_ESM.pdf]

Supplementary Information for

Milli-scale cellular robots that can reconfigure morphologies and behaviors simultaneously

Xiong Yang[1], RongTan[1], Haojian Lu[1,2], Toshio Fukuda[3], Yajing Shen[1,4,5*]

[1]Department of Biomedical Engineering, City University of Hong Kong, Hong Kong, China
[2]State Key Laboratory of Industrial Control and Technology, and Institute of Cyber Systems and Control, Zhejiang University, Hangzhou 310027 China
[3]Department of Micro-Nano Systems Engineering, Nagoya University, Nagoya, Japan
[4]Shenzhen Research Institute of City University of Hong Kong, Shenzhen 518057, China
[5]Department of Electronic and Computer Engineering, The Hong Kong University of Science and Technology, Clear Water Bay, Kowloon, Hong Kong, China
[*]Correspondence to: Yajing Shen (eeyajing@ust.hk)

Supplementary Figures

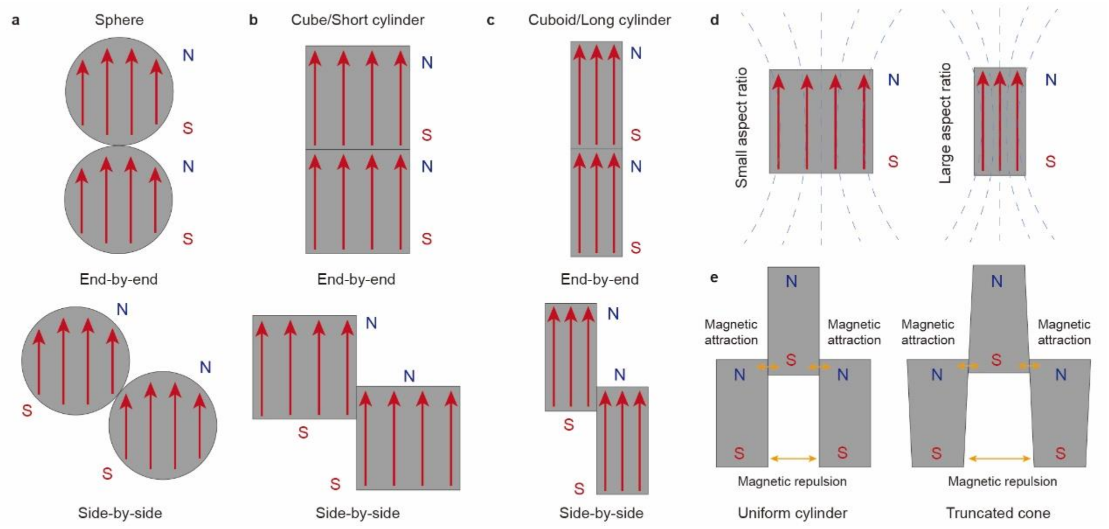

Supplementary Figure 1. **Design of mCEBOT units. a** The end-by-end and side-by-side connection of sphere units under the action of magnetic field is undifferentiated. **b** The end-by-end and side-by-side connection of cube or short cylinder units under the action of magnetic field. Where its small aspect ratio is not sensitive to the applied magnetic field, which may cause ineffective actuation and unexpected magnetization direction. **c** The end-by-end and side-by-side connection of cuboid or long cylinder units under the action of magnetic field. Considering the magnetic repulsion between same poles, this is not the most optimal shape. **d** The optimized cuboid/long cylinder can meet both the differentiated connection and directional magnetization due to the large aspect ratio. **e** Truncated cone design can endow unequal arrangements gap for reducing internal magnetic repulsion which enables the easier and more stable connection than the uniform cylinder with the same size.

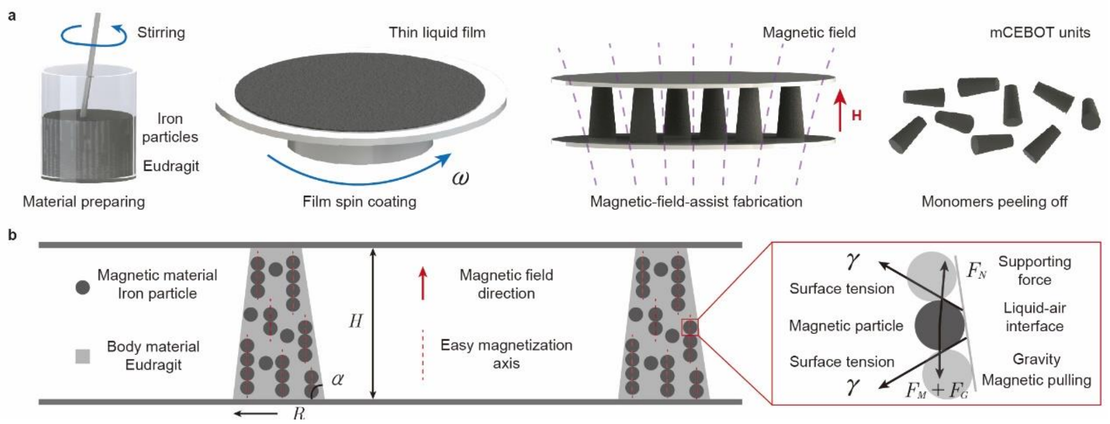

Supplementary Figure 2. **Fabrication process of mCEBOT units. a** At first, the raw material is prepared by mixing the iron particles and Eudragit solution. Then, the obtained raw material is coated on the non-magnetic plane following cover a top non-magnetic plane with the desired distance. After that, a vertical magnetic field is applied and the frustum shaped units will grow from the bottom substrate to the top substrate. Finally, the designed units are obtained by extracting them from the plate after curing. **b** Benefiting from the magnetic-field-assist manufacturing, the iron particles inside the unit are well aligned along its long axis to form the magnetic chains coinciding with the direction of the applied magnetic field lines. As the mechanical analysis shown the frustum shape is achieved under the combined action of magnetic field, surface tension and gravity. During which, the surface tension is related to the maximum achievable height and taper of mCEBOT unit. Under the same magnetic field, the larger surface tension will lead to larger taper and smaller achievable height of mCEBOT unit.

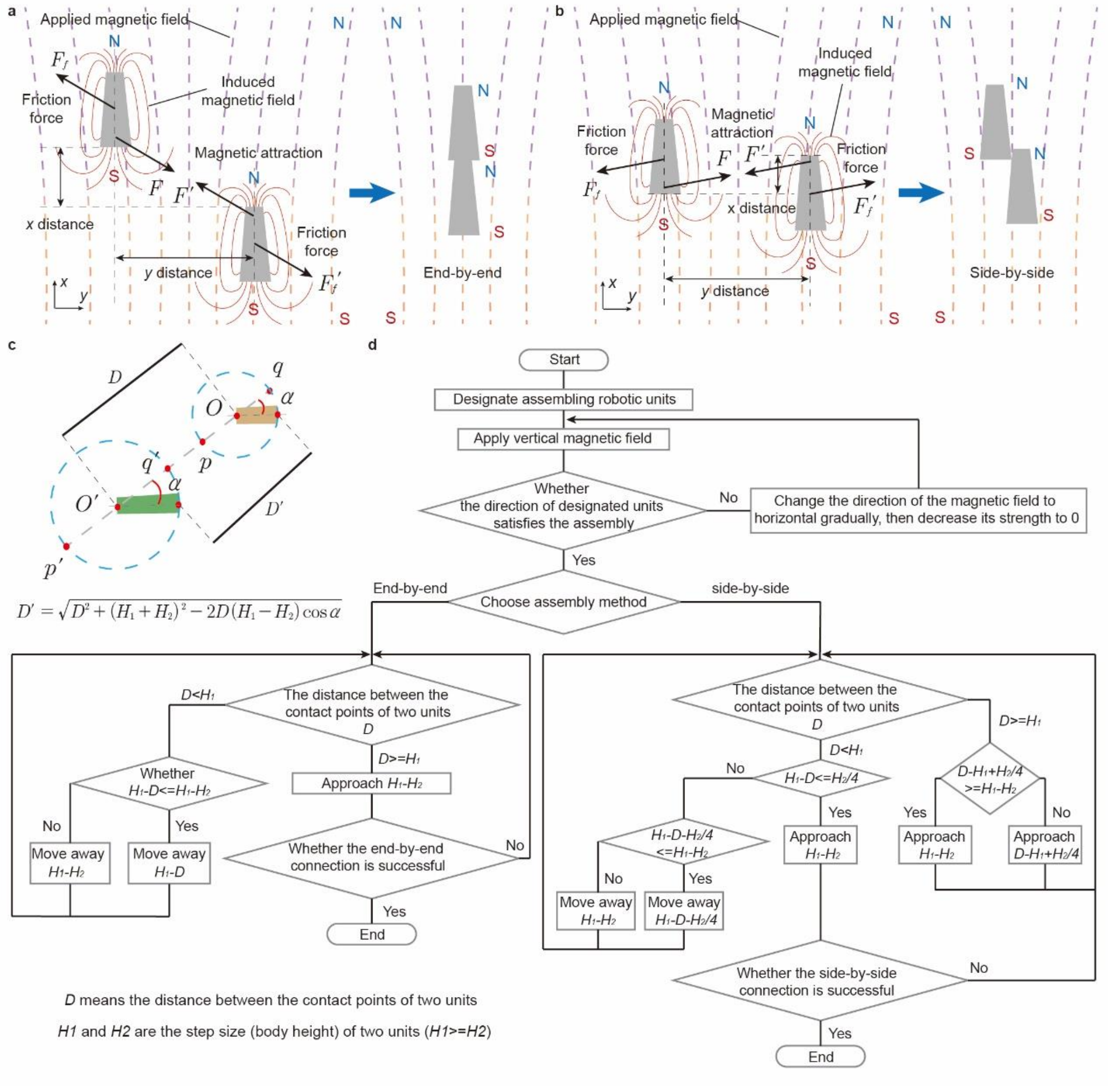

Supplementary Figure 3. **Analysis and strategy of assembly and separation. a** The mechanical analysis of end-by-end connection between two units under the external magnetic field. **b** The mechanical analysis of side-by-side connection between two units under the external magnetic field. **c** The state of long and short units during assembling, where their geometric relationship is defined by several parameters. **d** The detailed assembly strategy for single short and single long units, which can be extended to the assembly of universal units.

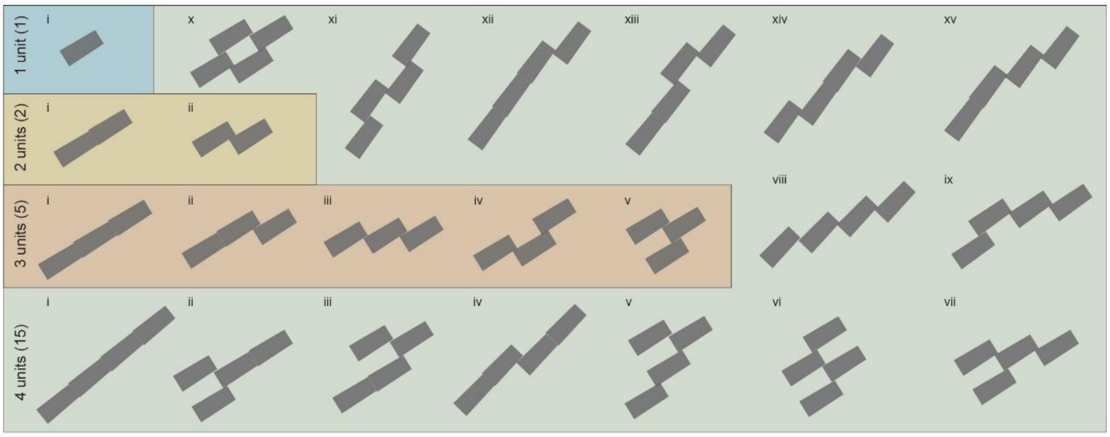

Supplementary Figure 4. **Expandability of mCEBOT with different number of units.** Protean architectures achieved by introducing different number of units and combining two connection methods.

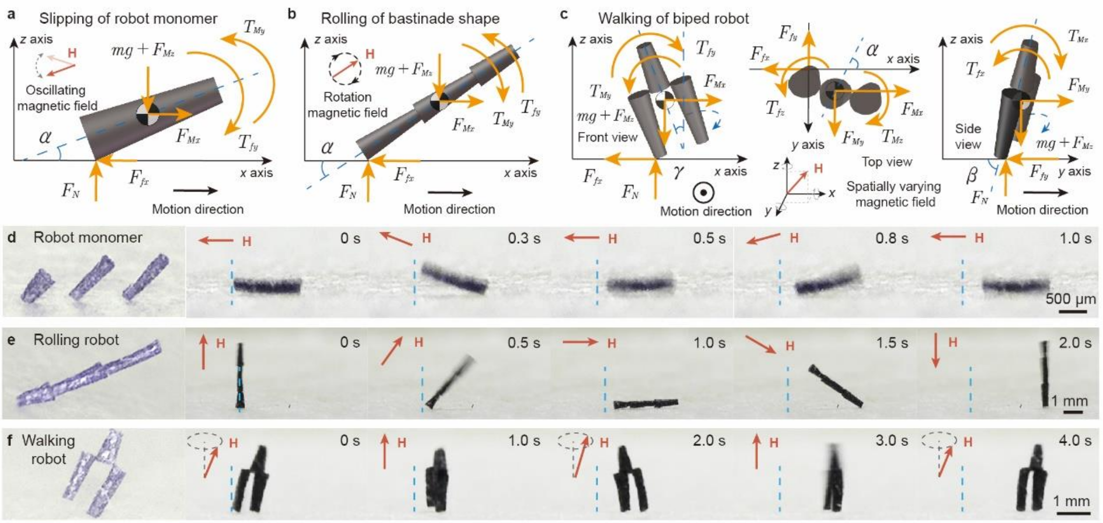

Supplementary Figure 5. **Analysis of slipping, rolling and walking. a** The mechanical analysis of slipping under the action of oscillating magnetic field. **b** The mechanical analysis of rolling under the action of rotating magnetic field. **c** The mechanical analysis of walking under the action of spatially varying magnetic field. **d** Slipping of robot monomer in separated state under oscillating magnetic field. **e** Rolling of bastinade shaped mCEBOT as a whole unit under rotating magnetic field. **f** Walking of biped robot in assembled state under the magnetic field with spatial variation of direction.

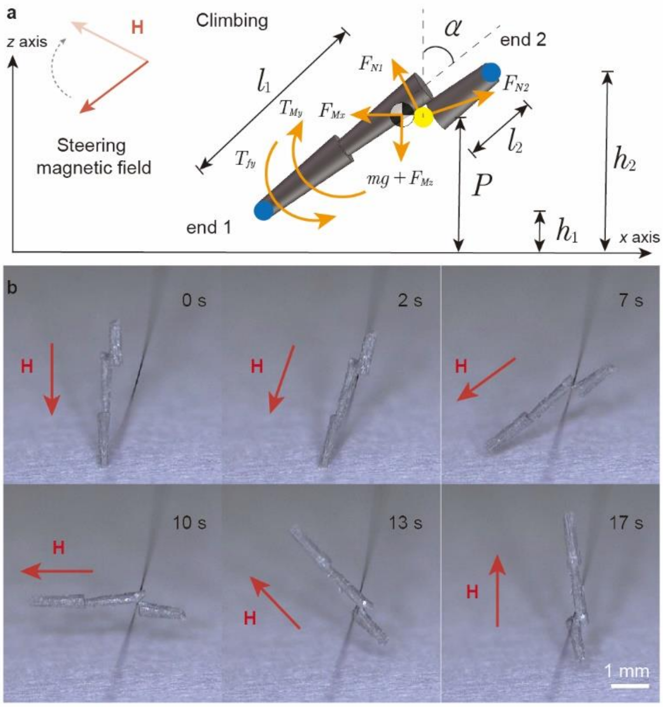

Supplementary Figure 6. **Analysis of climbing. a** The mechanical analysis of hoe shaped mCEBOT in *xz* plane, which is defined by tilt angle α, height of pivot *P*, distance from two ends to pivot l_1 and l_2, height of two ends h_1 and h_2, magnetic pulling force F_{Mx} and F_{Mz}, magnetic torque T_{My}, resistance torque T_{fy}, gravity *mg*, and support force F_{N1} and F_{N2}. **b** Climbing of hoe shaped mCEBOT as a whole unit under steering magnetic field by anchoring on vines-like pivot as fulcrum.

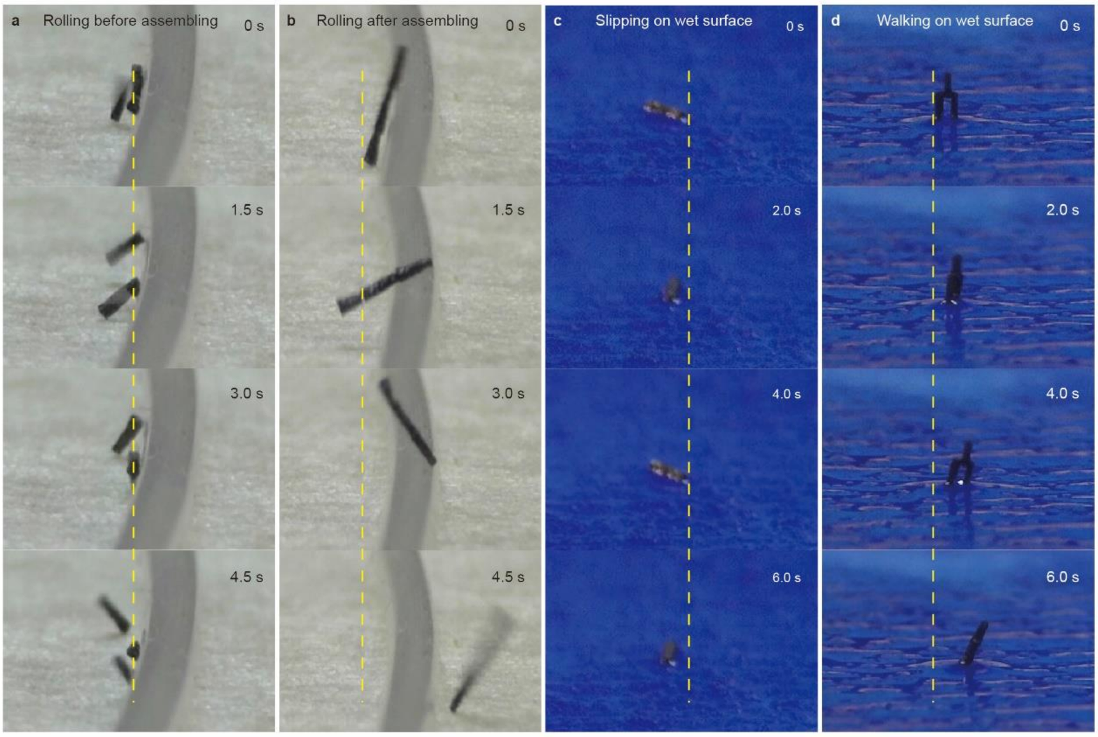

Supplementary Figure 7. **Performance comparison between different motion behaviors. a** mCEBOT units can not overcome the obstacle before assembling due to the height difference and slippery surface. **b** After assembling, the separated units are converted into the bastinade shaped mCEBOT, where its overall height increases greatly and makes the same obstacle overcoming easy. **c** The motion of mCEBOT in wet surface is still challenging due to the viscosity and tension from surface, and the inevitable line contact in slipping or rolling. To provide enough driving force and prevent unintended separation, a stronger magnetic field should be applied. **d** The resistance from surface tension can be greatly reduced benefited from the alternated point contact of biped structured mCEBOT.

Supplementary Notes

Supplementary Note 1: Design of mCEBOT units

The characteristic of robotic units will greatly influence the performance of constructed modular robot. Since the soft magnetic material and magnetic field are adopted to achieve the easy assembly and separation attributes, the size and shape should be considered during the design of robotic units. As Supplementary Figure 1a shown, despite the sphere shaped unit can achieve end-by-end and side-by-side connection under the magnetic field, its fully radially symmetric shape makes these two connection methods undifferentiated. The cube/short cylinder shape in Supplementary Figure 1b can enable the differentiated connection, but its small aspect ratio is not sensitive to the applied magnetic field which may cause ineffective actuation and unexpected magnetization direction. The optimized cuboid/long cylinder (Supplementary Figure 1c and 1d) can meet both the differentiated connection and directional magnetization due to the large aspect ratio. However, considering the magnetic repulsion between the same poles, the compact size of robotic units is restricted. To solve these problems, we proposed the truncated cone (frustum shape) as the mCEBOT unit. Comparing with the cuboid, the curved surface of cylinder allows higher fault tolerance which is more suitable for the practical in uneven ground or harsh environments. As shown in Supplementary Figure 1e, our truncated cone design can endow unequal arrangements gap for reducing internal magnetic repulsion which enables the easier and more stable connection than the uniform cylinder with the same size.

Supplementary Note 2: Analysis of the actuation ability of mCEBOT units

The mCEBOT unit will experience magnetic force and torque under the external magnetic field. Assuming the single magnetic particle is a point-like dipole with a magnetic susceptibility χ under the magnetic field **B**, R is the diameter of the spherical magnetic particle. For a specific mCEBOT unit, N is the total contained magnetic particles. Then, the endured magnetic force of mCEBOT unit can be expressed as: $F = N \bullet \frac{\pi R^3}{6} \bullet \chi \bullet \nabla B$. During which, the total number of magnetic particles N and the external magnetic field gradient ∇B are adjustable, which determine the magnetic force, i.e. the magnetic force of mCEBOT unit is proportional to the magnetic particle mass fraction and applied magnetic field. Since the magnetic particles inside the unit are aligned into magnetic chain during the curing process, the mCEBOT unit will endure magnetic torque until the magnetic field consists with the direction of magnetic chain. Assuming the magnetic chains are distributed evenly, d is the distance between inter particles, n is the number of the magnetic particles in a single chain, α is the angle between the direction of magnetic field and magnetic chain. Then, the total magnetic torque of the mCEBOT unit can be expressed as[1]: $T = N \frac{4\mu_0 n \chi^2 R^6 \pi}{3d^3} B^2 \sin 2\alpha$. For a specific unit, n is proportional to N, and d is proportional to $\frac{1}{N}$, i.e. the magnetic torque of mCEBOT unit is proportional to the magnetic particle mass fraction, applied magnetic field **B** and the phase angle α.

Supplementary Note 3: The calculation of actual generated magnetic force and torque

Since the magnetization of mCEBOT unit can be measured by the VSM and its volume can be estimated according to the shape as well as size, the generated magnetic force and torque can be calculated. For the mCEBOT unit with 30% magnetic particle mass fraction, its magnetization **M** under 100 mT magnetic field is about 230.8 KA/m. The volume of mCEBOT unit can be expressed as:

$$V = \frac{1}{3}\pi\left[R^3\tan\alpha - \frac{\left(R\tan\alpha - L\right)^3}{\tan^2\alpha}\right] \tag{1}$$

where L is the body length of unit (850 μm for short unit and 1200 μm for long unit), R=200 μm is the bottom radius, α =83° is the bottom angle. Then, the maximum generated magnetic force and torque of long unit and short unit under the magnetic field (strength 100 mT and gradient 4750 mT/m) can be calculated:

$$F_{short} = V_1 \bullet M \bullet \nabla B = 6.6\times10^{-2}\,mN \tag{2}$$

$$T_{short} = V_1 \bullet M \bullet B \bullet \sin(\frac{\pi}{2}) = 1.4\times10^{-6}\,N \bullet m \tag{3}$$

$$F_{long} = V_2 \bullet M \bullet \nabla B = 7.2\times10^{-2}\,mN \tag{4}$$

$$T_{long} = V_2 \bullet M \bullet B \bullet \sin(\frac{\pi}{2}) = 1.5\times10^{-6}\,N \bullet m \tag{5}$$

Supplementary Note 4: Analysis of mCEBOT units' assembly and separation

The assembly and separation of mCEBOT units are achieved by applying and removing magnetic field respectively. When the external magnetic field is applied, the units will be magnetized since the contained magnetic particles, and distribute their magnetic poles at two ends due to the frustum shape design. Further, the magnetized units will generate an induced magnetic field which results in the magnetic attraction between the

nearest opposite magnetic poles of neighboring units. When the magnetic attraction is larger than the friction force, the two units will approach and connect by end-by-end or side-by-side. During which, the strength of magnetic attraction under the unchanged external magnetic field is positive to the distance between opposite magnetic poles, and the force direction is related to the relative direction between opposite magnetic poles. As a result, the connection achievability and state are mainly dependent on the relative position between two opposite magnetic poles. Through experiments and statistics, the distribution of assembly state between two identical long units under 100 mT magnetic field is shown in Fig. 2b, Supplementary Figure 3a and 3b. Here, we define the distance between two neighboring units in long axis direction (horizontal distance) as x and the distance perpendicular to long axis (vertical distance) as y. We find that the neighboring units tend to assemble side-by-side when they have an overlapping part in horizontal distance, i.e. x is negative, and tend to assemble by end-by-end when horizontal distance x is positive. Since the soft magnetic property that we mentioned before, the magnetic attraction between units will disappear as the magnetic strength decreases to 0. Further, by following an oscillation magnetic field with decreasing strength (from 30 mT to 0 mT), the influence of electrostatic force can be got rid of and the separating process can achieve more effectively.

Supplementary Note 5: Assembly strategy

The conscious and selective assembly is achieved based on the different step size between units. As the diagram shown in Supplementary Figure 3c is the state of long and short units during assembling, where their geometric relationship is defined by

several parameters, including the height H_1 of long unit, the height H_2 of short unit, the distance D between the corresponding contact points of two units, the new distance D' between the next corresponding contact points of two units, and the actuation angle α. The flow chart shown in Supplementary Figure 3d is the detailed assembly strategy for short and long units. At first, we have to designate two specific units for assembling and apply a vertical magnetic field to actuate them to perpendicular to the ground. If the direction of designated units does not satisfy the assembly, then changing the direction of magnetic field to horizontal gradually and decrease its strength to 0. After that, applying the vertical magnetic field to actuate designated units to perpendicular to the ground again and loop this process until their directions satisfy the requirement of assembly. For the end-by-end connection, if the distance D is less than H_1 and the value $H_1 - D$ is less than or equal to the maximum step difference $H_1 - H_2$, then we will actuate the units to roll and make their distance between new contact points increase $H_1 - D$ until D is greater or equal to H_1. If the distance D is less than H_1 and the value $H_1 - D$ is greater than the maximum step difference $H_1 - H_2$, then we will actuate the units to roll and make their distance between new contact points increase $H_1 - H_2$ until D is greater or equal to H_1. After the distance D is greater or equal to H_1, we will actuate the units to roll and make their distance between new contact points decrease $H_1 - H_2$. And the above process will repeat until the designated end-by-end connection is successful. While for the side-by-side connection, if the distance D is greater or equal to H_1, we will actuate the units to roll and make their distance between new contact points decrease $H_1 - H_2$ (when

$D-H_1+H_2/4$ is greater or equal to H_1-H_2) or $D-H_1+H_2/4$ (when $D-H_1+H_2/4$ is less than H_1-H_2) until the distance D is less than H_1. After that, the value of H_1-D and $H_2/4$ will be compared. If the value H_1-D is greater than $H_1/4$, we will actuate the units to roll and make their distance between new contact points increase H_1-H_2 (when $D-H_1+H_2/4$ is greater or equal to H_1-H_2) or $H_1-D-H_2/4$ (when $D-H_1+H_2/4$ is less than H_1-H_2) until the judgment condition is invalid, then return to the value comparing between D and H. If the value H_1-D is less than or equal to $H_1/4$, we will actuate the units to roll and make their distance between new contact points decrease H_1-H_2. And the above process will repeat until the designated side-by-side connection is successful. During which, the directions of designated units can not only be adjusted randomly by applying and removing magnetic field repeatedly, but also be reversed by getting the help from obstacles' assistance. Fortunately, benefit from the high position errors tolerance ability of magnetic connection, the relative position requirement between units for assembly is greatly reduced, which increases the success rate of effective assembly in the practical. Although this flow chart is based on the assembly of single short and single long units, it can be extended to the assembly of universal unit. Noted that, the surface conditions and obstacles should be considered in the practical actuation of units which may makes the assembly process more complex. For example, when the harsh terrain is only in specific areas, it can be circumvented by adjusting the assembly paths. For the harsh terrain that is unavoidable, path and position errors can be compensated during subsequent movements. Besides that, the next step size of unit is not always equal to

the height body or constant, especially for the assembled unit which's connection point may not locate at the two ends, the distance between connection point and contact point should be analyzed and discussed according to the actual situation.

Supplementary Note 6: Slipping of individual mCEBOT unit

The slipping motion of robot monomer is achieved by the cooperation of force and torque from the oscillating magnetic field. As mechanical analysis shown in Fig. 4a and Supplementary Figure 5a, the independent robot monomer will align its long axis following the direction of magnetic flux under the action of magnetic torque. Combining with the action of gravity, one end of the robot monomer will keep contact with ground while the other end will be lifted. Further, when an oscillating magnetic field is applied, the independent robot monomer will swing with the applied magnetic flux direction and its two ends will contact the ground alternately. With the decrease of contact angle α in swing process, the friction resistance f_x will also decrease since the downward acceleration leads to smaller support force F_N. When f_x is smaller than the horizontal magnetic pulling force F_{Mx}, the independent robot monomer will slip forward driven by an instantaneous acceleration (Supplementary Figure 5d). Suppose the oscillating magnetic field in the *xz* plane and the robot monomer moves along *x* axis, then we can develop its dynamic model:

$$m\ddot{x}_{COM} = F_{Mx} - F_f \tag{6}$$

$$m\ddot{z}_{COM} = F_N - F_{Mz} - mg \tag{7}$$

$$J_C\ddot{\alpha} = T_M + F_f \frac{H}{2}\sin\alpha - F_N \frac{H}{2}\cos\alpha \tag{8}$$

where α is the tilt angle between robot monomer and *x* axis, J_C is the polar moment of inertia of the robot monomer, F_{Mx}, F_{Mz}, the pulling force of magnetic field along *x*

and z axis, F_N, the supporting force from the ground, F_f, the force of friction, mg is the gravity of robot, T_M , magnetic moment, T_f , resistance torque. *D, d, H* are the diameters of two ends and length of the robot monomer, where D is approximately equal to d and the $J_C \approx m\left(\left((D+d)/2\right)^2 + H^2\right)/12$.

Suppose the initial coordinate of point P (P_x, P_y) that is a contact point between robot monomer and ground, then the position and acceleration of the COM of robot monomer can be expressed as[2]:

$$x_{COM} = P_x + \frac{H}{2}\cos\alpha \tag{9}$$

$$z_{COM} = P_z + \frac{H}{2}\sin\alpha \tag{10}$$

$$\ddot{x}_{COM} = \ddot{P}_x - \frac{H}{2}\cdot\ddot{\alpha}\cdot\sin\alpha - \frac{H}{2}\cdot\dot{\alpha}^2\cdot\cos\alpha \tag{11}$$

$$\ddot{z}_{COM} = \ddot{P}_z + \frac{H}{2}\cdot\ddot{\alpha}\cdot\cos\alpha - \frac{H}{2}\cdot\dot{\alpha}^2\cdot\sin\alpha \tag{12}$$

By combining the equations (6)-(8) and (11)-(12), we have five equations and six unknown quantities $\left(\ddot{x}_{COM}, \ddot{z}_{COM}, \ddot{\theta}, \ddot{P}_x, \ddot{P}_z, F_f\right)$. To solve the under-defined system analytically, we first assume the contact point meets the pinned assumption, i.e. $\ddot{P}_x = 0$. If the solution shown $F_N < 0$ which means the contact point is not pinned, we should resolve the equations with $F_N = 0$, $F_f = 0$ and $\ddot{P}_x$ as an unknown. If the solution shown $F_f > F_{f\max}$ which also means the contact point is not pinned, we should resolve the equations with $F_f = F_{f\max}$ and $\ddot{P}_x$ as an unknown.

<u>Supplementary Note 7: Rolling of bastinade shaped mCEBOT</u>

For the bastinade shaped mCEBOT, it's not suitable to adopt the slipping motion because the bastinade shape may be disassembled due to the moving shock caused by the oscillating

magnetic field. To guarantee the smooth and stable movement of the bastinade shaped mCEBOT, we adopt a rolling motion pattern by applying the continuously rotating magnetic field. As illustrated in Fig. 4b and Supplementary Figure 5b, when the magnetic field rotates, the bastinade shaped mCEBOT will also rotate around the contact point, since its easy magnetization axis tends to follow the magnetic field direction. During which, the net magnetic moment **M** of mCEBOT is always along its long axis, when there is an angle between the net magnetic moment and magnetic field direction, the generated magnetic torque force under **B** can be expressed:

$$\tau = \mathrm{M} \times \mathrm{B} \tag{13}$$

On the one hand, this torque can actuate mCEBOT moving forward when magnetic field rotates in one direction. On the other hand, it can also steer the motion direction of mCEBOT by changing the rotation direction of magnetic field.

Based on that, the bastinade shaped mCEBOT can roll forward by adopting its two ends as fulcrum alternately under the action of a continuously rotating magnetic field. The dynamic equations of bastinade shaped mCEBOT rolling are similar to the robot monomer slipping. Different from the slipping discussed above, during rolling motion, the magnetic pulling force provides the downforce to enlarge the support force F_N, which can prevent the robot from unexpected skidding due to the induced large and stable friction resistance f_x. Moreover, the rotating magnetic field for rolling is smooth, unidirectional and continuous, which makes the rolling motion more controllable. For the bastinade shape with a length of l under the rotating magnetic field in *xz* plane with a frequency of f, assuming the rolling satisfies the no-slip condition, then the movement distance

L of the COM of mCEBOT in time t can be calculated as:

$$L = l\left\lfloor \frac{2t}{f} \right\rfloor + \left(\frac{l}{2} \cdot \left(1 - \cos\left(\left(\frac{2t}{f} - \left\lfloor \frac{2t}{f} \right\rfloor \right)\pi \right) \right) \right) \quad (14)$$

Where $\lfloor\ \rfloor$ means returning the integer part of the result. And the motion state in ½ gait cycle is shown in Supplementary Figure 5e.

Supplementary Note 8: Walking of biped structured mCEBOT

Since the magnetization of individual robotic unit along its long axis, the overall easy magnetization axis of assembled biped structured mCEBOT also tends to align with its long axis. Theoretically, this overall easy magnetization axis of robot will coincide with the magnetic field direction and tend to move to the local minimum[3]. Based on that, we apply the spatially varying magnetic field to convert the leg spacing of biped structured mCEBOT into effective step size. As the mechanical analysis shown in Fig. 4c and Supplementary Figure 5c, we define the biped structured mCEBOT's attitude by the angles between its long axis and three axes, which are α, β and γ corresponding to *x*, *y* and *z* respectively. At the initial state, the magnetic field is upright and the biped mCEBOT will stand up with two feet contact with ground. When the applied magnetic field has an angle γ' with the z axis in *xz* plane, there will be a torque T_{My} along the *y* axis to tilt the body of biped structured mCEBOT and lift one foot with the other foot as the fulcrum. Here, the torque equation can be expressed as:

$$MB\sin(\gamma' - \gamma) = mgl \cdot \sin\gamma \quad (15)$$

where M means the magnetization of assembled mCEBOT, **B** is the external magnetic field, mg represents the gravity of robot and l is the distance between the center of gravity and the bottom of the feet. Then, the actual tilt angle γ of mCEBOT with *z*

axis can be calculated as:

$$\gamma\text{=arctan}\left(\frac{MB\sin\gamma'}{mgl+MB\cos\gamma'}\right) \tag{16}$$

After that, we adjust the magnetic direction to exert extra torque T_{Mx} and T_{Mz} to make the biped mCEBOT leaning forward. Under the combined action of magnetic torque and gravity, the robot body will rotate angle ω around the fulcrum to convert the leg spacing into effective step size, and continuous walking can be achieved by alternating two feet as the fulcrum. During which, the step size of biped mCEBOT is related to its leg spacing S and rotate angle ω. When the biped mCEBOT walks under the spatially varying magnetic field with a frequency f, then the average velocity can be expressed as:

$$v = 4\cdot\frac{S\cdot f}{2}\sin\frac{\omega}{2} \tag{17}$$

The motion state of walking in one gait cycle is shown in Supplementary Figure 5f.

Supplementary Note 9: Climbing of mCEBOT under hoe mode

For the hoe shape of mCEBOT, the only one side-by-side assembly will form a hook due to the unaligned axes of units. When a pivot is in the reachable height of the hook, mCEBOT can anchor on the pivot as a frum and lift its whole body off the ground by climbing. As the mechanical analysis shown in Supplementary Figure 6a, when the hook anchoring on the pivot, the magnetic pulling force and gravity will make mCEBOT keep contact with pivot. When the magnetic field steers, the magnetic torque will work on mCEBOT and rotate it around the pivot since the overall easy magnetization axis of mCEBOT always tend to coincide with the magnetic field direction. Here we define the height of pivot as *P*, the tilt angle of mCEBOT as α, the

distance from two ends to pivot as l_1 and l_2 respectively. Suppose the hoe shaped mCEBOT work in in *xz* plane, then the height changing of two ends of mCEBOT can be expressed as:

$$h_1 = P - l_1 \cos\alpha \tag{18}$$

$$h_2 = P + l_2 \cos\alpha \tag{19}$$

The motion state of climbing under steering magnetic field is shown in Supplementary Figure 6b.

Supplementary Note 10: Characteristics analysis of motion behaviors in different morphologies

The locomotion of mCEBOT is not set randomly but configured according to its architectures to maximize the advantages. Despite the diverse achievable morphologies, they can be classified into four typical configurations, i.e., independent monomer, bastinade shape, biped structure and hoe shape. For the robot monomer in separated state, as the smallest indivisible modular unit, it has a smaller dimension and performs great superiority in narrow space tasks comparing with other assembled states. To keep the advantages endued by the tiny dimension, the slipping motion is adopted for the robot monomer by applying an oscillating magnetic field. For mCEBOT with bastinade shape, it achieves the maximum height by the end-by-end connection, which shows the potential advantages in obstacle overcoming and efficient movement. To make the utmost of the height of bastinade shape, the rolling motion is adopted by applying a rotating magnetic field. For the biped robot, comparing with the single robot monomer and bastinade shape, it is difficult to find the advantages in dimension. However, its bipedal locomotion with two legs likes human, can greatly reducing the resistance from surface viscosity and tension. For the hoe shape, the unaligned part of side-by-side assembly can work as hook for anchoring pivot. With the help of the external pivot, mCEBOT under the hoe mode

can reach a high place that is even higher than its own body length by climbing. As the experimental results shown in Supplementary Figure 7a and 7b, we first compare the effectiveness of slipping and rolling for overcoming the same obstacle. Benefiting from the advantages in dimension, mCEBOT in assembled bastinade shape can successfully overcome the high obstacle but doesn't work in separated state. Then, we compare the effectiveness of slipping and walking on wet surface (Supplementary Figure 7c and 7d). Due to the viscosity and tension from liquid surface, and the inevitable line contact in slipping, a stronger magnetic field should be applied to provide enough driving force. While the walking motion of biped mCEBOT is point contact, which is more effective than the other two types of locomotion patterns under the same magnetic field.

Supplementary References

1. Kim, J., Chung, S. E., Choi, S. E., Lee, H., Kim, J., & Kwon, S. Programming magnetic anisotropy in polymeric microactuators. Nat. mater., 10(10), 747-752 (2011).

2. Pawashe, C., Floyd, S., & Sitti, M. Modeling and experimental characterization of an untethered magnetic micro-robot. Int. J. Robot. Res., 28(8), 1077-1094 (2009).

3. Li, J., Wang, H., Shi, Q., Zheng, Z., Cui, J., Sun, T., ... & Fukuda, T. Biped walking of magnetic microrobot in oscillating field for indirect manipulation of non-magnetic objects. IEEE T. Nanotechnol., 19, 21-24 (2019).
