# Supplementary material for: Milli-scale cellular robots that can reconfigure morphologies and behaviors simultaneously
Source: Nat Commun. 2022 Jul 18;13:4156. doi: 10.1038/s41467-022-31913-0 (PMC9293897; doi:10.1038/s41467-022-31913-0)
Supplement: Supplementary file 3 — Description of Additional Supplementary Files [file 41467_2022_31913_MOESM3_ESM.pdf]

Description of Additional Supplementary Files

Supplementary Movie 1. Assembly, separation and reconfiguration of mCEBOT.
Supplementary Movie 2. Different motion behaviors of mCEBOT.
Supplementary Movie 3. Locomotion adaptability in complexed environment.
Supplementary Movie 4. mCEBOT for environment exploration and path marking.
